# The Impact of COVID-19 Lockdown on the Incidence of Type 1 DM and the Glycemic Control of Diabetic Children: Findings from a Teaching Hospital, Saudi Arabia

**DOI:** 10.1900/RDS.2022.18.152

**Published:** 2022-09-30

**Authors:** Mohammad Hussain Al-Qahtani, Fatimah Mousa Bukhamseen, Aqilah Taleb Al-Qassab, Abdullah Abdulsalam Yousef, Bassam Hassan Awary, Waleed Hamad Albuali, Zainab Mohammed Alkhalifa, Haneen Abdulsalam Yousef

**Affiliations:** 1 Department of Pediatrics, College of Medicine, Imam Abdulrahman Bin Faisal University, Dammam, Saudi Arabia,; 2 College of Medicine, Imam Abdulrahman Bin Faisal University, Dammam, Saudi Arabia,; 3 Pediatric Endocrine Fellow, Eastern Health Cluster, Dammam, Saudi Arabia,; 4 Department of Family Medicine, College of Medicine, Imam Abdulrahman Bin Faisal university, Dammam, Saudi Arabia.

**Keywords:** coronavirus, SARS-Cov-2 virus, COVID-19, type I diabetes mellitus, glycemic control, teaching hospital

## Abstract

**OBJECTIVE:**

We evaluated glycemic control among T1DM pediatric patients attending the endocrinology pediatrics clinics at King Fahd Hospital of the University (KFHU) prior to and during COVID-19 restraining regulations. In addition, we assessed the trends and variations in the incidence of T1DM during 2017-2021, including the COVID-19 years by identifying newly diagnosed patients presenting to pediatrics emergency department (ED) in KFHU.

**METHODS:**

To estimate the effect of COVID-19 on the incidence of T1DM, we identified newly diagnosed cases of T1DM among pediatric patients attending the ED during the years 20172021. The participants’ data were collected through electronic medical records. Information collected included patient age, sex, and HbA1c readings. Three HbA1c readings of interest that were defined and collected are pre-COVID reading, in-COVID reading, and post-COVID reading.

**RESULTS:**

The difference of female participants’ readings was statistically non-significant (Z= -0.416, p = 0.678), with a pre- and post-COVID median of 10.70 (Q1= 9.00, Q3= 12.15), and 10.50 (Q1= 8.80, Q3= 12.35), respectively. In contrast, the difference was statistically significant among male participants (Z= -2.334, p = 0.02), with a pre- and post-COVID median of 10.20 (Q1= 8.70, Q3= 11.80), and 10.65 (Q1= 9.00, Q3= 12.70), respectively. There was a statistically significant increase in HbA1c of persons > 11 years old (Z= -2.471, p= 0.013), with a pre- and post-COVID median of 10.40 (Q1= 9.00, Q3= 12.10), and 10.90 (Q1= 9.00, Q3= 12.60), respectively. Conversely, persons ≤ 11 years old showed no statistically significant change in HbA1c (Z= -.457, p= 0.648), with a pre- and post-COVID median of 10.45 (Q1= 8.70, Q3= 11.85), and 10.20 (Q1= 8.40, Q3= 12.075), respectively. Disregarding any influence of time, the effect of sex showed no statistically significant difference in HbA1c between males and females [F (1,125) = 0.008, p = 0.930]. Meanwhile, the age effect on HbA1c, regardless of time influence, was statistically significant [F (1,125) = 4.993, p = 0.027]. There was no statistically significant interaction between time and sex on HbA1c levels [F (1.74, 217) = 0.096, p = 0.883] and between age and time [F (3.92,289.57) = 1.693, p = 0.190].

**CONCLUSIONS:**

The number of visits to healthcare facilities dropped significantly during the COVID-19 pandemic, but the rate of newly diagnosed T1DM increased. There was a variable effect on HbA1c levels of those patients, which suggests that each demographic group in the population might have been affected differently by the pandemic. Future research should determine factors associated with better glycemic control and measures to sustain these changes the pandemic might have created.

## 1 Introduction

Coronavirus disease 2019 (COVID-19) is a highly contagious infectious disease caused by the severe acute respiratory syndrome coronavirus 2 (SARS-Cov-2 virus). It is a respiratory disease with variable manifestations ranging from asymptomatic to mild symptoms to more severe illness. Globally, governments implemented many measures to avoid the spread of the virus, thereby minimizing the burden on their countries [1,2].

The pandemic has transformed daily lives and continues to disrupt the everyday routines of societies.

According to World Health Organization, the total number of confirmed cases are over 515 million worldwide, of which 755,415 have been reported in Saudi Arabia [3]; the Saudi government imposed a lockdown order on March 25, 2020.

In 2020, the incidence of COVID-19 was reported less among pediatric patients and contributed to 8% of the daily reported cases [4]. However, since late December 2021, a spike in the incidence and hospitalization in the pediatric age group was noticed, especially for younger children who are ineligible for vaccination [5].

As the pandemic persists, new challenges keep emerging in healthcare related to the dynamics of managing acute and chronic diseases. One of the most prevalent chronic conditions worldwide that affects a wide range of age groups is diabetes mellitus [6]. Saudi Arabia has documented remarkably high numbers of type 1 diabetes mellitus (T1DM) among children and adolescents that puts it in the lead among countries in the Middle East and North Africa, with an estimated prevalence of 27,800 in 2019 [7]. As the COVID-19 pandemic situation entailed governments to impose several social restrictions, including the lockdown, a persistent demand is rising to understand the impact of these actions on such a vulnerable group.

The influence of COVID-19 on the glycemic control of diabetic patients has been discussed in the literature; however, it remains inconclusive. Many overlapping factors have been identified that could influence blood glucose levels throughout these different periods. For instance, increased parent care, as well as the drop in consumption of fast food, resulted in a decline of glycated hemoglobin (HbA1c) [8,9]. On the other hand, a decreased level of physical activity and limited access to healthcare services along with other factors had a negative impact on blood glucose level [10].

As the incidence of T1DM grows, the impact of COVID-19 restrictions on this population should be thoroughly investigated to be able to mitigate any potential negative consequences. The purpose of the study is to evaluate and analyze the glycemic control among T1DM pediatric patients attending the endocrinology pediatrics clinics at King Fahd Hospital of the University (KFHU) prior to and during COVID-19 restraining regulations. In addition, we assess the trends and variations in the incidence of T1DM during 2017-2021, including the COVID-19 years by identifying newly diagnosed patients presenting to pediatrics emergency department in KFHU.

## 2 Methods

This is a longitudinal observational retrospective study, which took place in the diabetic pediatrics clinics and the emergency department at King Fahd Hospital of the University (KFHU), a teaching hospital in Al-Khobar, Saudi Arabia. To estimate the effect of COVID-19 on the incidence of T1DM, we identified newly diagnosed cases of T1DM among pediatric patients attending the emergency department (ED) during the years 2017-2021. In our facility, once a patient’s diagnosis is established, a unique problem code is assigned to the patient’s electronic medical record which corresponds to the predefined ICD-9 codes available in the master repository of the system. Accordingly, we were able to identify all individuals who we under 18 years of age and labeled as T1DM (ICD-9 code 250.01) to be included in this study. The number of ED visits per year was obtained retrospectively through the register, and it served as the dominator in establishing the incidence rate. Simultaneously, the target population to assess the effects of COVID-19 pandemic on the control of T1DM included pediatric patients ≥ 2 years of age but < 18 years diagnosed with T1DM, who had attended any pediatric diabetes clinic, between the months of September 2019 and March of 2020. There are 3 pediatric diabetic clinics per week in our facility, taking place at different days of the week. Patients diagnosed with diabetes and < 18 years old are referred to the clinic through the ED or after general pediatric ward admission. An average of 6.4 patients are seen in each clinic.

**Figure 1.**
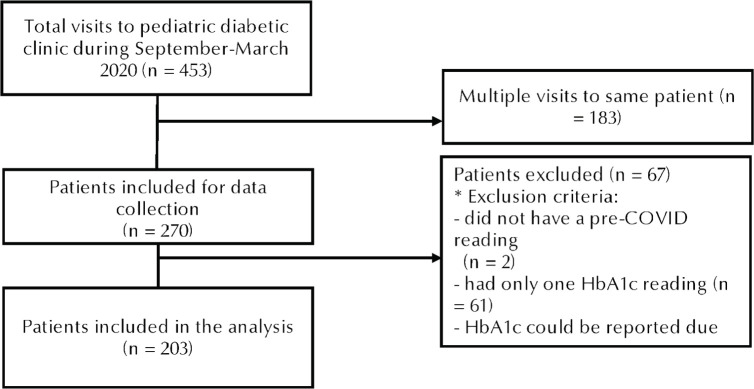
Flowchart of included participants.

The participants’ data were collected through electronic medical records. Information collected included patient age, sex, and HbA1c readings. Three HbA1c readings of interest that were defined and collected are pre-COVID reading, in-COVID reading, and post-COVID reading. The pre-lockdown reading is defined as any reading prior to lockdown to 3 months after the start of lockdown (March 9, 2020). When multiple pre-lockdown readings are available, the latest is considered. The in-lockdown reading is the earliest reading post 3 months from the start of lockdown. Finally, the post-lockdown reading is defined as the latest reading taken in 2021. Initially, a list of 270 patients who have visited the diabetic clinics between September 2019 and March 2020 were examined. Those who did not have a pre-COVID reading, had only one HbA1c reading, or whose HbA1c could not be reported due to a hemoglobin variant were excluded. A total of 203 participants were included in the analysis (Figure 1).

Data analysis was performed using the Statistical Package of Social Sciences (SPSS) version 26 (IBM Corp, Armonk, NY). Normality was examined using the Shapiro-Wilk test. Continuous variables were summarized as means ± standard deviation (SD) while categorical variables were summarized using frequency and percentages. A mixed ANOVA was used to test the statistical significance of main and interaction effects of the independent variables on the HbA1c levels of the participants. For skewed data, Wilcoxon signed-rank tests was used. A p < 0.05 was considered statistically significant.

## 3 Results

### 3.1 Effect of COVID on T1DM incidence

**Figure 2.**
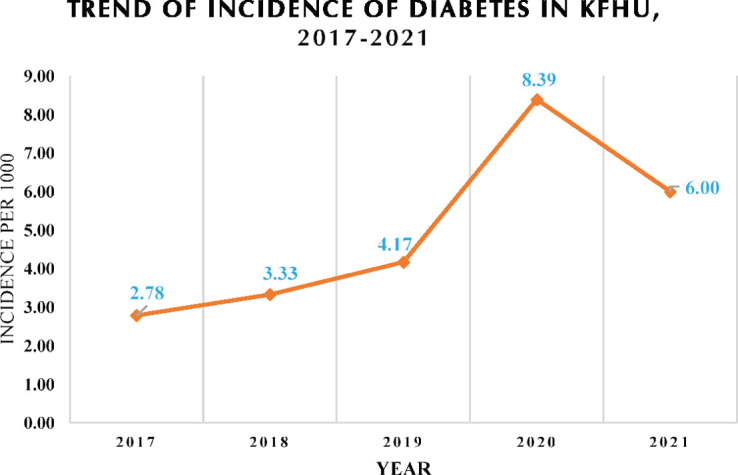
Trend of the annual incidence.

A total of 940 patients with newly diagnosed T1DM occurred during the years spanning the study. Table 1 shows the annual incidence rate throughout the years of 2017- 2021. Figure 2 shows the trend of the incidence rate.

**Table 1.** Annual incidence rate

Year	Cases at risk	New cases	Incidence rate	Incidence rate per 1000
2017	63932	178	0.002784208	2.78
2018	60090	200	0.003328341	3.33
2019	62367	260	0.004168871	4.17
2020	19906	167	0.00838943	8.39
2021	22505	135	0.005998667	6.00

### 3.2 Effect of COVID on HbA1c readings

Out of the 203 participants included in this study, 112 (55%) were females and 91(45%) were males. The predominant age group included those > 11 years old, with only 72 being ≤ 11 years old. HbA1c was recorded at 3-time intervals. To assess the influence of COVID lockdown on the glycemic control, the participants HbA1c was examined and analyzed using 2 different models.

#### 3.2.1 Two-point analysis

First, a 2-group analysis was done with all 203 participants included. The groups were sorted to pre-COVID readings and post-COVID readings, the latter including either in-lockdown or post-lockdown readings. The median pre-COVID and post-COVID HbA1c readings were 10.40 (Q_1_= 8.90, Q_3_= 11.90), and 10.50 (Q_1_= 8.90, Q_3_= 12.50), respectively. The results showed no statistically significant difference between pre- and post-COVID readings (Z= -1.759, p = 0.079). This was further analyzed by sex and age groups.

Wilcoxon signed-rank tests indicated that the difference of female participants’ readings was statistically non-significant (*Z*= -0.416, p = 0.678), with a pre- and post-COVID median of 10.70 (Q_1_= 9.00, Q_3_= 12.15), and 10.50 (Q_1_= 8.80, Q_3_= 12.35), respectively. In contrast, the difference was statistically significant among male participants (Z= -2.334, p = 0.02), with a pre- and post-COVID median of 10.20 (Q_1_= 8.70, Q_3_= 11.80), and 10.65 (Q_1_= 9.00, Q_3_= 12.70), respectively.

Among the age groups, Wilcoxon signed-rank tests revealed a statistically significant increase in HbA1c readings of those aged > 11 years old (Z= -2.471, p= 0.013), with a pre- and post-COVID median of 10.40 (Q_1_= 9.00, Q_3_= 12.10), and 10.90 (Q_1_= 9.00, Q_3_= 12.60), respectively. Conversely, those ≤ 11 years of age showed no statistically significant change in HbA1c readings (Z= -.457, p= 0.648), with a pre- and post-COVID median of 10.45 (Q_1_= 8.70, Q_3_= 11.85), and 10.20 (Q_1_= 8.40, Q_3_= 12.075), respectively.

#### 3.2.2 Three-point analysis

Data were then analyzed at 3 chronological points. The mean readings of pre-lockdown, in-lockdown, and post-lockdown periods were 10.5074 (SD = 2.21654), 10.5818 (SD = 2.52848), and 10.5817 (SD = 2.41831), respectively. Females had higher readings overall, except for the post-lockdown period where males displayed higher HbA1c readings. As per the age distribution, those older than 11 years had a tendency toward higher HbA1c readings (Table 2).

**Table 2.** Mean HbA1c readings per gender and age distribution

	Pre-COVID HbA1c	In-COVID HbA1c	Post-COVID HbA1c
Males	10.4278	10.5561	10.6442
Females	10.5708	10.6024	10.5280
< 11 years	10.440	10.071	10.243
> 11 years	10.544	10.892	10.755

While disregarding any influence of time, the effect of sex was analyzed using a mixed ANOVA test, which showed no statistically significant difference in HbA1c between males and females [F (1,125) = 0.008, p = 0.930]. Meanwhile, the age effect on HbA1c readings, regardless of time influence, was statistically significant [F (1,125) = 4.993, p = 0.027]. The main effect of time on HbaA1c levels was analyzed while adjusted for both sex and age in parallel fashion, and both results were non-significant [sex: F (1.74, 217) = 1.86, p = 0.30; age: F (1.25,289.57), p = 0.559]. Likewise, there was no statistically significant interaction between time and sex on HbA1c levels [F (1.74, 217) = 0.096, p = 0.883] and between age and time [F(3.92,289.57) = 1.693, p = 0.190].

## 4 Discussion

Over the course of the COVID-19 pandemic during the last 2 years, several papers have discussed its effect on diabetes development and control. In Saudi Arabia, there was an increase in the incidence of T1DM among pediatric patients during the pre-pandemic period [11,12]. However, these local trends have not been described and analyzed compared to the pandemic period.

In our longitudinal observational retrospective study, we measured the annual incidence rate for 3 years prior to the COVID-19 pandemic and 2 years during the pandemic, revealing higher annual incidence rate in our institution during the COVID-19 outbreak – from 4.17 in 2019 to 8.39 and 6.00 per 1000 during 2020 and 2021, respectively.

Our results were consistent with international studies conducted to assess the incidence of pediatric T1DM during the COVID-19 pandemic and any changes in the pattern of development. A multicenter regional study in Northwest London that involved 5 in-patients centers reported a total number of 30 new-onset cases during April/May 2020 with an increase in 2 centers, but a constant rate in the other 3 centers [13]. Similarly, a cross-sectional study in San Diego collected data over a 12-month interval throughout the in-COVID period and compared their results with the annual incidence prior to COVID-19 pandemic. Higher rates of newly diagnosed T1DM were reported compared to the previous year, 189 and 119 respectively [14].

Meanwhile, many authors have speculated that the COVID-19 pandemic has had an influence on diabetic patients’ glycemic control. The lockdown situation in particular has created new circumstances for all populations that directly affect patients’ ability to control their well-being. However, literature concerning T1DM patients’ glycemic control is controversial. Several studies have concluded that the lockdown had a positive influence on glycemic control. The Fernandez et al study demonstrated a substantial improvement in HbA1c readings, particularly in those with a baseline higher than 8% [15]. Another study among Danish children attending diabetic clinics showed comparable results of HbA1c reduction in those with a baseline > 7.5%. Nevertheless, they also found those with a baseline HbA1c < 6.9% to have a marginal increase in their readings. In contrast to our finding of age being a factor influencing poor glycemic control regardless of time, they found that those older than 11 years demonstrated a significant reduction in HbA1c over time [16].

In comparison, other studies found that glycemic control measures became worse after the lockdown period. A Saudi study revealed that the lockdown was associated with a significant increase in the mean blood glucose level (200.45 ± 79.970) in comparison with prelockdown period (182.09 ± 76.68) [10]. This finding aligns with Hosomi et al.’s study that described a significant worsening of HbA1c readings among T1DM patients in Kyoto, Japan [17]. Similar to our results, a study conducted at an urban pediatric teaching hospital in Texas found that increasing age was associated with higher HbA1c readings, although there was no difference between pre- and post-lockdown groups [18].

Our study has several limitations, because of its retrospective nature, not all glycemic control measures could be assessed. In addition, our findings are based on data collected from a teaching center, that serves an urban area. Therefore, it is possible that these data reflect the situation among those individuals for whom healthcare is more readily accessible. Furthermore, the pediatric endocrine clinics in our center were running virtually for almost 8 months during and after the lockdown, and the majority of patients tended to avoid attending healthcare centers in person to minimize exposure to COVID-19. This resulted in a lower rate of laboratory work-up for this population, and thus, limited the number of patients included in this study.

Our investigation was just a single center-based study so the results cannot be generalized to other settings; however, this encourages other large centers nationally as well as internationally to publish their results to determine many of the factors that might affect the incidence of T1DM and the glycemic control of diabetic children during the critical and unusual period of the COVID-19 era.

### 4.1 Conclusion

The number of visits to healthcare facilities dropped significantly during the COVID-19 pandemic; however, the rate of newly diagnosed T1DM markedly increased. The literature both locally and internationally is inconclusive concerning the influence of COVID-19 on glycemic control. In our study, the situation had a variable effect on HbA1c levels of those patients, which suggests that each demographic group in same population might have been affected differently by the pandemic. Future research should determine factors associated with better glycemic control and measures to sustain these changes the pandemic might have created. Furthermore, these findings might help healthcare providers plan for such exceptional situations to manage the T1DM children trying to keep their glycemic control on target levels.

## Abbreviations

COVID-19: Coronavirus disease 2019 SARS-Cov-2 virus Severe acute respiratory syndrome coronavirus 2

T1DM: Type 1 diabetes mellitus

KFHU: King Fahd hospital of the university

ED: Emergency department

SPSS: Statistical package for social sciences

SD: Standard deviation
